# Protein- and zinc-deficient diets modulate the murine microbiome and metabolic phenotype^1^,^2^

**DOI:** 10.3945/ajcn.116.131797

**Published:** 2016-10-12

**Authors:** Jordi Mayneris-Perxachs, David T Bolick, Joy Leng, Greg L Medlock, Glynis L Kolling, Jason A Papin, Jonathan R Swann, Richard L Guerrant

**Affiliations:** 3 Division of Computational and Systems Medicine, Department of Surgery and Cancer, Imperial College London, London, United Kingdom;; 4 University of Virginia Center for Global Health and; 5 Department of Biomedical Engineering, University of Virginia, Charlottesville, VA; and; 6 School of Veterinary Medicine, University of Surrey, Guildford, United Kingdom

**Keywords:** malnutrition, metabolome metabolic phenotype, metabolome metabonomics, microbiome microbiota, protein deficiency, undernutrition, zinc deficiency

## Abstract

**Background:** Environmental enteropathy, which is linked to undernutrition and chronic infections, affects the physical and mental growth of children in developing areas worldwide. Key to understanding how these factors combine to shape developmental outcomes is to first understand the effects of nutritional deficiencies on the mammalian system including the effect on the gut microbiota.

**Objective:** We dissected the nutritional components of environmental enteropathy by analyzing the specific metabolic and gut-microbiota changes that occur in weaned-mouse models of zinc or protein deficiency compared with well-nourished controls.

**Design:** With the use of a ^1^H nuclear magnetic resonance spectroscopy–based metabolic profiling approach with matching 16S microbiota analyses, the metabolic consequences and specific effects on the fecal microbiota of protein and zinc deficiency were probed independently in a murine model.

**Results:** We showed considerable shifts within the intestinal microbiota 14–24 d postweaning in mice that were maintained on a normal diet (including increases in Proteobacteria and striking decreases in Bacterioidetes). Although the zinc-deficient microbiota were comparable to the age-matched, well-nourished profile, the protein-restricted microbiota remained closer in composition to the weaned enterotype with retention of Bacteroidetes. Striking increases in Verrucomicrobia (predominantly *Akkermansia muciniphila*) were observed in both well-nourished and protein-deficient mice 14 d postweaning. We showed that protein malnutrition impaired growth and had major metabolic consequences (much more than with zinc deficiency) that included altered energy, polyamine, and purine and pyrimidine metabolism. Consistent with major changes in the gut microbiota, reductions in microbial proteolysis and increases in microbial dietary choline processing were observed.

**Conclusions:** These findings are consistent with metabolic alterations that we previously observed in malnourished children. The results show that we can model the metabolic consequences of malnutrition in the mouse to help dissect relevant pathways involved in the effects of undernutrition and their contribution to environmental enteric dysfunction.

## INTRODUCTION

Many parts of the developing world are still struggling with malnutrition, inadequate clean-water supplies, and a lack of access to basic health care. These conditions lead to a vicious cycle of malnutrition, infection, and environmental enteric dysfunction that cause growth stunting and cognitive shortfalls. To better understand the impact of nutritional restrictions on the health and development of an individual, murine models have been developed to experimentally mimic human malnourishing diets. These models have previously been shown to replicate the characteristic side effects of malnutrition including reduced infant growth, delayed neurobehavioral development, and permanent alterations in macrophage function (1, 2). In this work, we have investigated 2 malnourishing mouse diets that reflected major nutritional deficiencies that are common in children who are living in impoverished areas. These diets included a protein-deficient diet (containing 2% protein; a normal diet typically contains ∼20% protein) that resulted in growth failure and a diet that was devoid of zinc that led to zinc deficiency compared with that shown in zinc-deficient children.

These murine models provided a tool for dissecting the biochemical mechanisms through which specific nutritional restrictions can lead to phenotypic outcomes. Metabolic phenotyping (metabolomics and metabonomics) is a systems biology approach that enables the overall metabolic status of a biological system to be studied. The combination of metabolic phenotyping with specific models of undernutrition provides a top-down approach to illuminate the metabolic pathways that are modulated by such restrictions. A key variable that influences the metabolic fate of dietary inputs and, indeed, are shaped by the diet are the gut microbiota. Previous studies have shown that malnutrition can alter the structure and function of the bacterial populations that are present in the gut with subsequent alterations to the nutrient flow to the host (3–5). The importance of this biochemical interchange between the microbiota and host has been widely appreciated with known downstream consequences for host endogenous metabolism and, ultimately, host health. Therefore, to adequately resolve the biomolecular impact of a malnourishing diet and its influence on development, the bacterial-host supraorganism was studied including the diverse and complex *trans*-genomic metabolic interactions that occur (5, 6). To this extent, the urinary metabolic profiles and fecal bacterial populations of mice receiving either a protein-deficient or zinc-deficient diet have been characterized and compared with well-nourished control mice. Urinary metabolic profiles contain information relating to the host endogenous metabolism and also exogenous metabolic inputs such as those from the diet and the output from the gut microbiome. Through this parallel approach, the impact of malnourishing diets on the overall mammalian system can be resolved, thereby allowing the diet-associated mechanisms that underlie the phenotypic outcomes in the host to be understood.

## METHODS

### Animal studies

Male 22-d old C57BL/6 mice were provided by Jackson Laboratories. Mice weighed ∼11 g on arrival and were co-housed in groups of ≤5 animals/cage. The vivarium was kept at a temperature between 20°C and 24°C with a 14-h light and 10-h dark cycle. The study was carried out in strict accordance with the recommendations in the Guide for the Care and Use of Laboratory Animals of the NIH. The protocol was approved by the Committee on the Ethics of Animal Experiments of the University of Virginia (protocol 3315).

### Rodent diets

**FIGURE 1:**
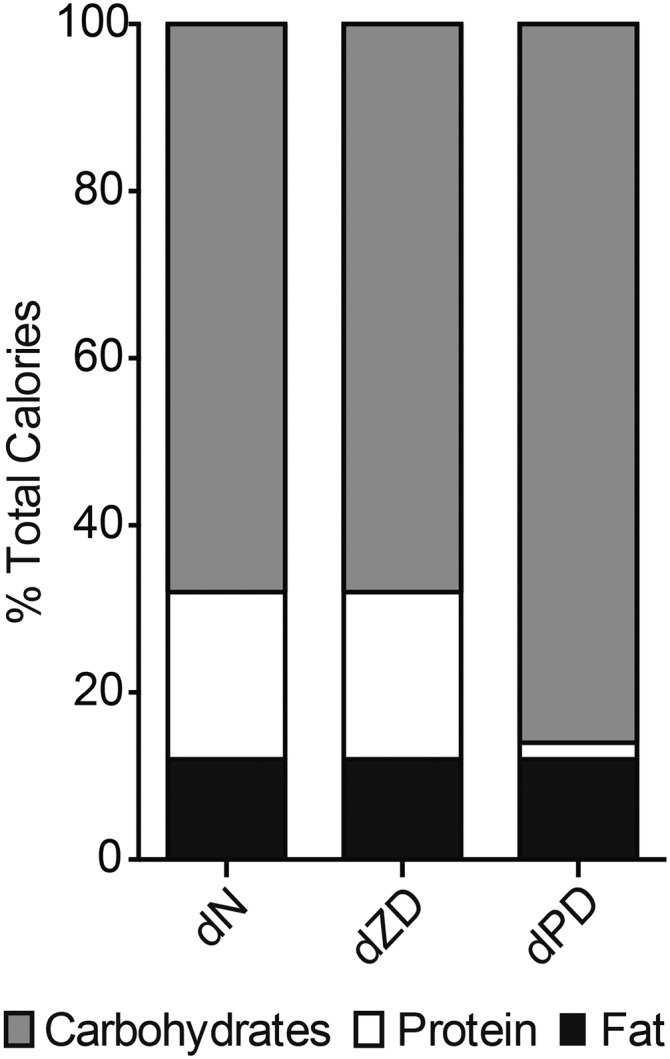
Mean ± SEM percentages of calories from fat, protein, and carbohydrates of the isocaloric diets used in the study. dN, defined normal; dPD, defined protein deficient; dZD, defined zinc deficient.

Weaned mice (22 d old) were randomly assigned to experimental groups and fed either a protein source–defined normal (dN)^7^ diet (*n* = 10; containing 20% protein) or a defined protein-deficient (dPD) diet (*n* = 10; containing 2% protein) for 14 d (aged 36 d the end of study). A defined zinc-deficient (dZD) diet (<2 ppm zinc, 20% protein; *n* = 8) was provided for 10 d to 36-d-old mice that were maintained on the dN diet for 14 d postweaning (46 d old at the end of the study) and were compared with age-matched, well-nourished equivalents (dN diet for 24 d; 0.056 g Zn and 20% protein; *n* = 10). A 14-d acclimatization period with the dN diet was necessary for the dZD mice because of the severity of outcomes that arise from zinc deficiency directly from weaning. Diets were obtained from Research Diets. Calories from fat, protein, and carbohydrates are shown in **Figure 1**. All diets were isocaloric, and complete formulations are provided in Supplemental Table 1.

### Lipocalin-2 and myeloperoxidase measurements

After 10–14 d of consumption of the diet, stools were collected from the mice for the measurement of lipocalin-2 and myeloperoxidase. Samples were homogenized in a radioimmunoprecipitation assay buffer with protease inhibitors and centrifuged at 8000 × *g* for 10 min at room temperature, and the supernatant fluid was collected. The stool supernatant fluid was assayed for total protein (bicinchoninic acid assay), lipocalin-2, and myeloperoxidase (R&D Systems) according to the manufacturer’s instructions. Data were expressed as pg lipocalin-2 or myeloperoxidase/μg total protein.

### DNA isolation and amplification

DNA was isolated from fecal pellets with the use of the QIAamp DNA Stool Mini Kit as previously described. The V3–V4 hypervariable regions of the *16S ribosomal RNA* gene from fecal DNA samples were amplified with the use of specific primers (Illumina; forward: 5′-TCGTCGGCAGCGTCAGATGTGTATAAGAGACAGCCTACGGGNGGCWGCAG–3′, reverse: 5′–GTCTCGTGGGCTCGGAGATGTGTATAAGAGACAGGACTACHVGGGTATCTAATCC–3′).

### 16S sequencing and data analysis

The 16S libraries were pooled and sequenced with the use of the MiSeq Reagent Kit v3 that produces 25 million reads of 2 × 300 bp/run at the Genomics Core Facility at the University of Virginia. Reads were assigned to samples with the use of BaseSpace demultiplexing (Illumina). From these reads, the bacterial presence and relative abundance were quantified with the use of the QIIME package (version 1.9.1) (7). Fastq-join was called via QIIME to join paired-end reads with a minimum of a 6-bp overlap and 8% maximum difference (8). Barcodes were extracted from paired reads, and reads were quality filtered with the use of split_libraries.py from QIIME with default variables. Chimeric sequences were detected and removed with the use of reference-based and de novo chimera identification with USEARCH61 (9) and the GreenGenes16S ribosomal RNA database (10). The identification of operational taxonomic units (OTUs) was performed by referencing the GreenGenes database (http://greengenes.lbl.gov/cgi-bin/nph-index.cgi) with UCLUST (97% sequence identity cutoff) and de novo OTU picking with QIIME. The Ribosomal Database Project classifier was used to assign taxonomy to identified OTUs. The weighted UniFrac distance (11) between each sample was calculated, and a principal coordinates analysis (PCoA) was performed on the resulting distance matrix. PCoA results were visualized with EMPeror (12). To prepare OTU data for the comparison of the relative abundance of bacterial genera between dietary conditions, the relative abundance of each OTU was determined, and OTUs were filtered according to the following 2 criteria: being present in ≥2 samples and having a 0.5% relative abundance in ≥1 sample. With the use of the remaining OTUs and their relative abundances, a Kruskal-Wallis test was performed to identify OTUs that were significantly overrepresented or underrepresented in ≥1 condition (MATLAB and Statistics Toolbox Release 2015b; The Mathworks Inc.). OTUs that had a Bonferroni-corrected *P* < 0.05 were considered significantly different in ≥1 condition. A post hoc Dunn-Sidak multiple-comparisons test was performed on all OTUs that passed the filtering criteria with the assumption that OTUs with *P* < 0.05 were significantly different between conditions.

### ^1^H nuclear magnetic resonance spectroscopy–based metabolic profiling

Urine samples were analyzed with the use of ^1^H nuclear magnetic resonance (NMR) spectroscopy. Each sample was prepared by combining 30 μL urine with 30 μL phosphate buffer (pH 7.4; 100% D_2_O) that contained 1 mmol internal standard/L, 3-trimethylsilyl-1-[2,2,3,3-^2^H_4_] propionate (TSP). Samples were mixed with the use of a vortex and centrifuged (10,000 × *g*) for 10 min at room temperature before transfer to a 1.70mm NMR tube. A spectroscopic analysis was performed on a 700-MHz Bruker NMR spectrometer equipped with a cryoprobe. Standard one-dimensional ^1^H NMR spectra of the urine samples were acquired with water-peak suppression with the use of a standard pulse sequence. For each sample, 8 dummy scans were followed by 128 scans and collected in 64-K data points. A recycle delay of 2 s, a mixing time of 10 μs, and an acquisition time of 3.8 s were used. The spectral width was set at 20 ppm. Chemical shifts in the spectra were referenced to the 3-trimethylsilyl-1-[2,2,3,3-^2^H_4_] propionate singlet at δ = 0.0.

Spectra were manually phased and corrected for baseline distortions. ^1^H NMR spectra (δ = 0.2–10.0) were digitized into consecutive integrated spectral regions (∼20,000) of equal width (0.00055 ppm). The regions between δ = 4.50 and 5.00 were removed to minimize the effect of baseline effects that were caused by imperfect water suppression. Each spectrum was normalized to the unit area. A multivariate modeling was performed in MATLAB with the use of in-house scripts. The modeling included a principal components analysis with the use of paretoscaling and an orthogonal projection to latent structures discriminant analysis (OPLS-DA) that was constructed with the use of unit-variance scaling. OPLS-DA models were constructed to assist the model interpretation. ^1^H NMR spectroscopic profiles were used as the descriptor matrix, and class membership (e.g., nourished and protein malnourished) was used as the response variable. Correlation coefficients plots were generated with the use of back scaling transformation to display the contribution of each metabolite to the sample classification. The color scale represented the significance of a correlation for each metabolite to the class membership with red indicating strong significance and blue indicating weak significance. The predictive performance (Q^2^Y) of the model was calculated with the use of a 7-fold cross-validation approach, and the model validity was established with the use of permutation testing (1000 permutations).

### Clustering analysis

An unsupervised hierarchical clustering analysis (HCA) was performed to identify general patterns of biochemical and microbial variations between samples. For metabolome data, unsupervised clustering for all samples was done with the use of the normalized abundance of metabolites that were identified through the OPLS-DA models. To prepare the OTU data, the relative abundance of each OTU was determined, and OTUs were filtered according to the following 2 criteria: being present in ≥2 samples and having a 0.5% relative abundance in ≥1 sample. Metabolites that were identified as contributing to the separation between diets through the OPLS-DA models were used for sample clustering. For comparison purposes, only metabolites that were present in all diets were used. OTUs that were present in ≥2 samples, had a 0.5% relative abundance in ≥1 sample, and identified to be significantly overrepresented or underrepresented in ≥1 diet were used for sample clustering. For a comparative analysis across different metabolites and OTUs, data were standardized as *z* scores across samples for each metabolite and OTU before clustering so that the mean was 0 and the SD was 1. This standardized matrix was subsequently used in unsupervised HCA for samples and metabolites or OTUs with the use of correlation-based distances (Pearson correlation) from which hierarchical clusters were generated with the use of an average-linkage method by means of the pdist and linkage functions in the MATLAB bioinformatics toolbox. Heat maps and dendrograms after the HCA were generated with MATLAB imagesc and dendrogram functions, respectively. In the heat maps, a red-blue color scale was used whereby shades of red and blue represented higher and lower values, respectively, compared with the mean. Different diet groups were color coded and shown under the dendrogram for each sample.

### Metabonome and microbiota correlation analysis

To explore the functional correlation between the gut microbiota changes and metabonome perturbations, a Spearman correlation analysis between metabolites and the OTU abundances was performed. The Benjamini-Hochberg method was used to adjust *P* values for multiple testing with consideration of a 5% false-discovery rate.

### Statistical analysis

Unless otherwise stated, data analyses were performed with GraphPad Prism 5 software (GraphPad Software). All statistical analyses were performed from raw data with the use of an ANOVA (growth-curve data), Student’s *t* tests, and a Bonferroni post hoc analysis (myeloperoxidase and lipocalin-2 data) when applicable. Differences were considered significant at *P* < 0.05. Data are presented as means ± SEMs.

## RESULTS

### Phenotypic alterations after malnourishing diets

**FIGURE 2:**
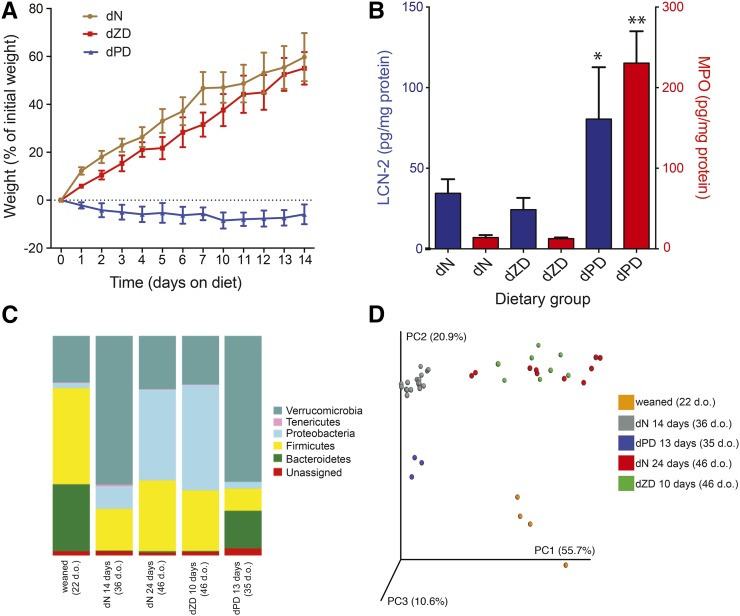
Mean ± SEM phenotypic and fecal microbial alterations after protein and zinc deficiency. (A) Growth comparison of mice from a separate experiment that were weaned to dN and dPD diets for 14 d (22 d.o. at the start of the diets). An additional growth curve is shown for 36-d.o. mice (weaned to a dN diet for 14 d) that were transferred to receive the dZD diet for 14 d *(n* = 5/group). Significant differences in growth were observed between dPD and dN mice (*P* < 0.001) after being fed the diets for 1 d as determined with the use of a Students *t* test. (B) Amounts of LCN-2 (blue bars) and MPO (red bars) in the stool after 10–14 d of being fed the different experimental diets. *^,^**dPD compared with dN or dZD mice (Student’s *t* test): *P < 0.05, ** *P* < 0.001. (C) Phylum-level resident microbiota profiles after 10–14 d of being fed the diets. (D) Principal coordinate analysis of the weighted UniFrac distance of different diet groups [weaned (22 d.o.; *n* = 4), dN diet for 14 d (36 d.o.; *n* = 16), dPD diet for 13 d (35 d.o.; *n* = 3), dN diet for 24 d (46 d.o.; *n* = 10), and dZD diet for 10 d (46 d.o.; *n* = 8)]. dN, defined normal; d.o., days old; dPD, defined protein deficient; dZD, defined zinc deficient; LCN-2, lipocalin-2; MPO, myeloperoxidase; PC, principal coordinate.

Growth curves were plotted from a separate mouse study that followed the same protocol of feeding dN and dPD diets over a 14-d period postweaning and feeding the dZD diet for 14 d after 14 d consumption of the dN diet (**Figure 2A**). A significant growth deficit of ∼60% was observed in the mice fed the dPD diet (*n* = 5) compared with the dN controls (*n* = 5). Similar growth was observed between the mice fed the dN and dZD (*n* = 5) diets from 36 d of age. The growth response was consistent between these mice and those studied for microbiomic and metabolic analyses.

### dPD diet increased markers of intestinal inflammation

Stool samples were collected after 10–14 d of the diet, and lipocalin-2 and myeloperoxidase were measured with the use of an ELISA. Similar to the growth data, significant changes were only observed in mice fed the dPD diet, with elevated amounts of these markers of intestinal inflammation in the stool (Figure 2B). A 2-fold increase in lipocalin-2 was observed in mice fed the dPD diet compared with in those fed the dN diet (*P* = 0.038). However, a marked 20-fold increase in myeloperoxidase was observed in these animals compared with the dN mice (*P* = 0.033).

### Composition variation in the stool microbiota after malnourishing diets

Phylum-level shifts were identified in the fecal microbiota influenced by the dietary composition and length of time of the diet. No significant differences were observed in the total copies of 16S between groups as measured with the use of a quantitative polymerase chain reaction, which indicated that the bacterial abundance was consistent across all animals (data not shown). Figure 2C shows the phylum-level relative abundance of the fecal microbiota from mice maintained ≤24 d that were fed the selected diets. After weaning (22-d-old mice), decreasing amounts of Bacteroidetes and Firmicutes were observed in mice that were fed the dN diet for 14 d (36-d-old mice). In contrast, bacteria that belonged to Verrucomicrobia, Tenericutes, and Proteobacteria were observed to expand during this time period. After a further 10 d of the dN diet (46-d-old mice), the Verrucomicrobia and Tenericutes returned to abundances seen at weaning, whereas the Proteobacteria expanded further and the amount of Firmicutes increased. Similar phylotypes were observed between dN-fed mice (46 d old) and those fed the dZD diet for 10 d (46 d old). Mice that were fed the dPD diet for 13 d (35 d old) postweaning were shown to differ from similar-aged (36-d-old) mice that received the dN diet. A postweaning expansion in Verrucomicrobia was observed in the dPD fed mice as with the dN diets. However, the dramatic loss of Bacteroidetes that was seen with the dN diet was not observed with the dPD diet, and this phylum remained a notable proportion (17.1%) of the microbiota. In addition, the postweaning loss of Firmicutes was more pronounced with the dPD diet. No increase in Tenericutes was observed in these mice.

Both measures of Chao1 diversity and Faith’s phylogenetic diversity showed that weaned animals had the highest diversity followed by the dPD group and that dN and dZD groups had the lowest diversity measures regardless of the number of days that they were fed the diet (Supplemental Figure 1). This greater relative diversity in the weaned animals may have been due to the diverse and rich composition of milk received from lactating mice and the exposure to the maternal microbiota. As shown in Figure 2D, the PCoA of the weighted UniFrac distance from the data set showed a separation into color-coded clusters according to the diet. After the application of filtering criteria as described in Methods, statistical analyses were performed to determine differences in the relative abundance of bacterial genera between dietary groups (statistics are provided in Supplemental Table 2). Consistent findings were observed between the dPD samples with matched metabolic data and repeated microbiota analyses with the use of a larger sample size with increased sampling points (weaned: *n* = 16; dPD and dN: *n* = 8 each at 5, 8, 12, and 15 d of diet consumption) (Supplemental Figure 2).

### Metabolic disruptions induced by malnourishing diets

**FIGURE 3:**
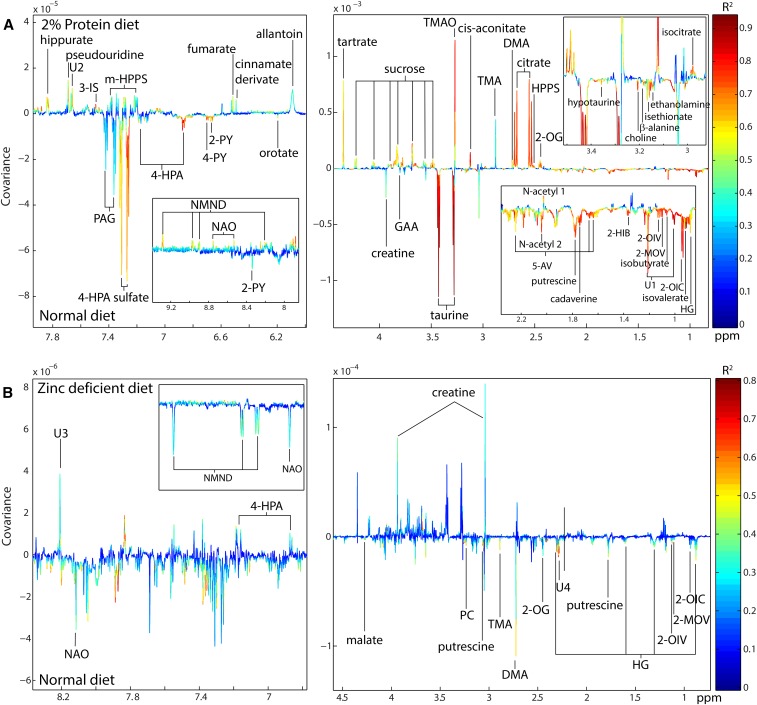
OPLS-DA models constructed from the urinary metabolic profiles of mice on different dietary regimens. OPLS-DA coefficients plots are shown that indicate the metabolic variation between protein malnutrition (A) (dPD: *n* = 10; dN: *n* = 16; Q^2^Y = 0.87, *P* = 0.001) and zinc deficiency (B) (dZD: *n* = *8*; dN: *n* = 8; Q^2^Y = 0.37, *P* = 0.016) compared with the respective well-nourished controls. ^1^H nuclear magnetic resonance spectroscopic profiles serve as the descriptor matrix and class membership (e.g., dN or dPD; dN or dZD) as the response variable. Correlation coefficients plots were generated with the use of a back-scaling transformation to display the contribution of each metabolite to the sample classification. Positive peaks indicate metabolites that were excreted in greater amounts with the malnourishing diets than with the dN diet, and negative peaks indicate metabolites that were excreted in lower amounts. The color scale represents the significance of the correlation for each metabolite to the class membership with red indicating strong significance and blue indicating weak significance. DMA, dimethylamine; dN, defined normal; dPD, defined protein deficient; dZD, defined zinc deficient; GAA, guanidinoacetic acid; HG, hexanoylglycine; m-HPPS, *m*-hydroxyphenylpropionylsulfate; NAO; nicotinamide-*N*-oxide; NMND, *N*-methylnicotinamide; OPLS-DA, orthogonal projection to latent structures discriminant analysis; PAG, *N*-phenylacetylglycine; PC, phosphocholine; ppm, parts per million; TMA, trimethylamine; TMAO, trimethylamine-*N*-oxide; U, unknown metabolite; 2-HIB, 2-hydroxyisobutyrate; 2-MOV, 3-methyl-2-oxovalerate; 2-OG, 2-oxoglutarate; 2-OIC, 2-oxoisocaproate, 2-OIV, 2-oxoisovalerate; 2-PY, *N*-methyl-2-pyridone-5-carboxamide; 3-IS, 3-indoxylsulfate; 4-HPA, 4-hydroxyphenylacetate; 4-PY, *N*-methyl-4-pyridone-3-carboxamide; 5-AV; 5-aminovalerate.

Pairwise OPLS-DA models were constructed to compare the age-matched urinary metabolic profiles of mice fed the malnourishing (dPD and dZD) diets and those fed the standard (dN) diet. A model with a strong predictive ability [Q^2^Y = 0.87; *P* = 0.001 (1000 permutations)] was obtained that compared the urinary spectral profiles of mice that received the dN diet for 14 d (*n* = 16; 36 d old) and the dPD diet for 13 d (*n* = 10; 35 d old). The metabolic alterations induced by the dPD diet compared with the dN diet are shown in the coefficient plot from this model in **Figure 3A** and are summarized in **Supplemental Figure 3** (^1^H NMR spectroscopy chemical shifts for each metabolite are provided in **Supplemental Table 3**). Mice that consumed a protein-deficient diet excreted greater amounts of tricarboxylic acid (TCA) cycle metabolites (citrate, *cis* aconitate, isocitrate, 2-oxoglutarate, and fumarate), metabolites from the nicotinate and nicotinamide pathway [*N*-methyl-nicotinamide and nicotinamide-*N*-oxide (NAO)], gut microbial metabolites of choline (dimethylamine and trimethylamine), gut microbial host cometabolites 3-indoxylsulfate, *m*-hydroxyphenylpropionylsulfate, hippurate, and trimethylamine-*N*-oxide), purine and pyrimidine metabolites (allantoin and pseudouridine), and the dietary constituents sucrose, tartrate, and cinnamate than did mice that were maintained with the dN diet. Mice that consumed the dPD diet excreted lower amounts of metabolites that arose from branched-chain amino acid (BCAA) catabolism [isovalerate, isobutyrate, 2-oxoisocaproate, 2-oxoisovalerate, and 3-methyl-2-oxovalerate (2-MOV)], polyamines (putrescine and cadaverine), glycine conjugates of β-oxidation intermediates (hexanoylglycine), taurine-related metabolites (taurine, hypotaurine, and isethionate), NAD-catabolism end products [*N-*methyl-2-pyridone-5-carboxamide (2-PY) and *N-*methyl-4-pyridone-3-carboxamide (4-PY)], creatine, guanidinoacetic acid, ethanolamine, β-alanine, choline, and orotate, and the gut microbial-derived metabolites 2-hydroxyisobutyrate, 5-aminovalerate, 4-hydroxyphenylacetate, and *N*-phenylacetylglycine than did mice fed the dN diet.

Significant differences were also shown when comparing the urinary metabolic profiles from mice that consumed the dZD diet for 10 d (*n* = 8; 46 d old) with their age-matched, nourished equivalents (*n* = 8; 46 d old) although to a lesser extent than was observed with the dPD diet [OPLS-DA Q^2^Y = 0.37; *P* = 0.016 (1000 permutations)] (Figure 3B, Supplemental Figure 3). After zinc deficiency, greater excretions of creatine and 4-hydroxyphenylacetate were observed, and lower excretions of hexanoylglycine, 2-MOV, 2-oxoisocaproate, 2-oxoisovalerate, putrescine, 2-oxoglutarate, dimethylamine, trimethylamine, phosphocholine, *N*-methylnicotinamide, and NAO were shown compared with in the nourished animals.

**FIGURE 4:**
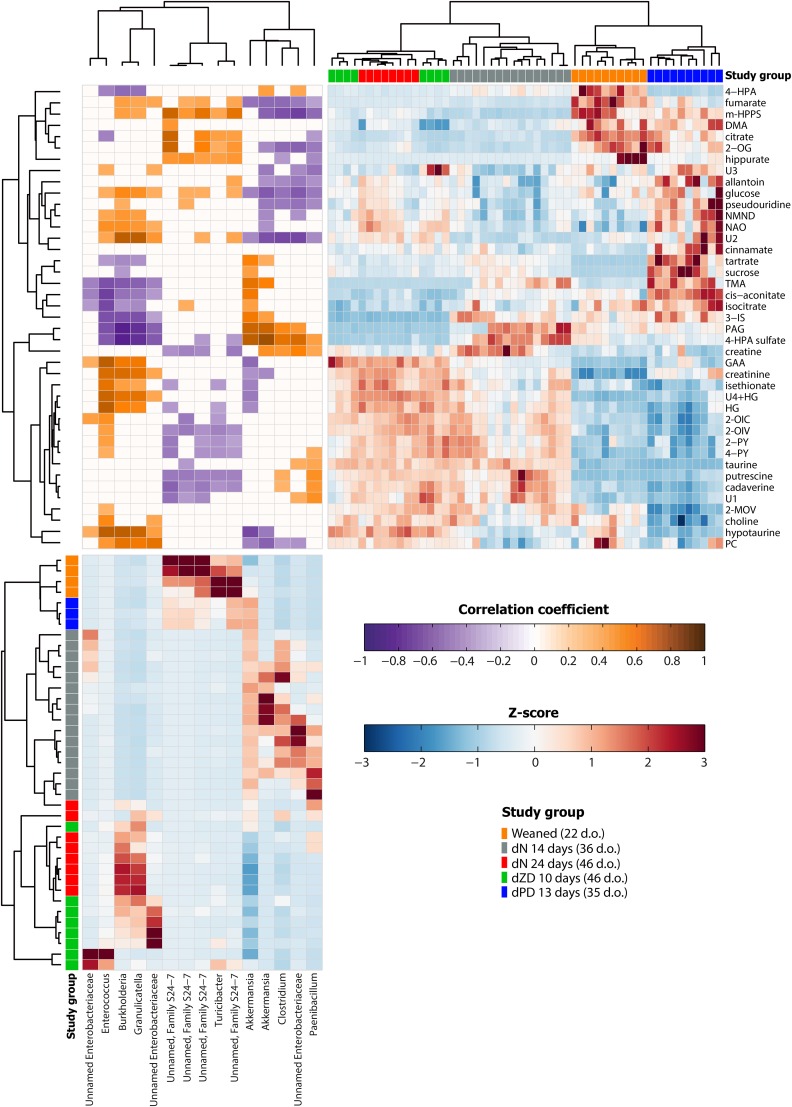
Unsupervised hierarchical clustering of the metabonome and gut bacteria for all mice. (Upper right quadrant) Metabolite clustergram displaying *z* scores of metabolites for each mouse. A metabolite *z*-score transformation was performed on the intensity of each metabolite across samples. Metabolites shown are those that were identified in orthogonal projection to latent structures discriminant analysis models as varying between study groups. Metabolites and samples are clustered by correlation distance and average-linkage hierarchical clustering. Study-group assignments are indicated by color bars [weaned (22 d.o.; *n* = 10); dN 14 d (36 d.o.; *n* = 16); dN 24 d (46 d.o.; *n* = 8); dPD (35 d.o.; *n* = 10); and dZD (46 d.o.; *n* = 8)]. (Lower left quadrant) Bacterial clustergram displaying *z* scores of specific bacteria for each mouse. Each row corresponds to an individual mouse, and each column corresponds to a specific OTU. OTUs selected were those that passed the filtering criteria as stated in Methods. An OTU *z*-score transformation was performed on the abundance of each OTU across samples. OTUs and samples were clustered by the correlation distance and average-linkage hierarchical clustering. Weaned: *n* = 4; dN at 14 d: *n* = 16; dN at 24 d: *n* = 8; dPD: *n* = 3; and dZD: *n* = 8. (Upper left quadrant) Correlation heat map derived with the use of Spearman's correlation to identify statistical linkages between OTUs and metabolites. The intensity of the colors represents the degree of correlation. Metabolites and OTUs were clustered by Euclidean distance and average-linkage hierarchical clustering. Only significant correlations after adjustment of *P* values (with the use of the Benjamini-Hochberg method for 5% false-discovery rate) are shown. DMA, dimethylamine; dN, defined normal; d.o., days old; dPD, defined protein deficient; dZD, defined zinc deficient; GAA, guanidinoacetic acid; HG, hexanoylglycine; m-HPPS, *m*-hydroxyphenylpropionylsulfate; NAO; nicotinamide-*N*-oxide; NMND, *N*-methylnicotinamide; OTU, operational taxonomic unit; PAG, *N*-phenylacetylglycine; PC, phosphocholine; TMA, trimethylamine; U, unknown metabolite; 2-MOV, 3-methyl-2-oxovalerate; 2-OG, 2-oxoglutarate; 2-OIC, 2-oxoisocaproate, 2-OIV, 2-oxoisovalerate; 2-PY, *N*-methyl-2-pyridone-5-carboxamide; 3-IS, 3-indoxylsulfate; 4-HPA, 4-hydroxyphenylacetate; 4-PY, *N*-methyl-4-pyridone-3-carboxamide.

A clustering analysis of the metabolic variation that was associated with dietary exposure is presented in **Figure 4** (upper right quadrant). From this plot, the urinary profiles of the study mice were shown to cluster by the diet and by the time consuming the diet. Consistent with the bacterial observations, the urinary profiles of the dPD mice were closer in composition to those of the weaned mice than of the other study animals. An age- and dietary duration–associated metabolic variation was observed in the dN-fed mice with the 36-d-old mice clustering separately from the 46-d-old mice. From this clustergram, the mice that received the dN diet for 24 d (46 d old) shared greater similarity with the dZD mice than with the younger mice that received the dN diet for 14 d (36 d old). This result suggests that an age-related biochemical variation was greater than that induced by the dZD diet.

### Microbial-metabolic interactions in response to malnutrition

From the clustergram constructed on the bacteria identified to be significantly different across nutritional groups (Figure 4, lower left quadrant), the bacterial composition of the dPD-fed mice was most similar to the samples collected from the weaned animals (22 d old) than to those of any other study group. Both weaned and dPD mice had higher abundances of unnamed family S24-7 than those of other study animals. After being fed the dN diet for 14 d, OTUs assigned to Clostridium, unnamed Enterobacteriaceae, and, in some mice, Paenibacillus were observed to increase from the weaned profile before decreasing after 24 d of the dN diet. Such increases were not observed with the dPD diet. *Akkermansia muciniphila* was observed to increase in all animals 14 d after weaning regardless of the diet before decreasing in dN or dZD mice 10 d later. No notable differences were observed in the bacterial phylotypes of 46-d-old mice that received the dN or dZD diet. An increase in OTUs assigned to Burkholderia and Granulicatella was observed in several animals from these groups compared with in the younger dN-fed animals (36 d old). The correlation heat map (Figure 4, upper left quadrant) identified significant correlations between OTUs and metabolites. *A. muciniphila* was positively correlated with the urinary excretion of *N*-phenylacetylglycine, 3-indoxylsulfate, trimethylamine, creatine, *cis* aconitate, sucrose, and tartrate. This bacterium was also negatively correlated with *m*-hydroxyphenylpropionylsulfate, glucose, fumarate, creatinine, guanidinoacetic acid, hypotaurine, phosphocholine, and hexanoylglycine.

## DISCUSSION

In this report, we characterized the metabolic perturbations and the gut microbial alterations induced by zinc or protein deficiency in the mouse. Consistent with the human condition, intestinal inflammation and growth shortfalls were observed in the mice that received the protein-restricted diet but not in those that were deprived of zinc. We have previously shown that the villus:crypt ratio was decreased by a dPD diet compared with a dN diet but not with a dZD diet (13, 14). These phenotypic modulations that were induced by protein deficiency were accompanied by pronounced differences in the development of the gut microbiota compared with that seen in well-nourished mice. The fecal microbial composition of protein-deficient mice was shown to more closely resemble that of mice at the point of weaning than of age-matched, well-nourished mice. In contrast, a minimal microbial variation was observed between well-nourished mice and those that were deficient in zinc. Note that the greatest diversity in the fecal microbiota was seen at the point of weaning with this diversity being partially maintained by the protein-deficient diet. The bacterial acquisition from the dam and a greater variety of nutrients in the breast milk than in the defined experimental diets, including of prebiotic oligosaccharides, were likely to have caused this increased bacterial diversity (15). We hypothesize that the lack of adequate protein in the dPD diet locked the microbiota into its premalnourished state. This phenomenon has also been reported in malnourished children (6). Although it is plausible that intestinal inflammation after protein deficiency could modulate the gut environment and change the bacterial composition, it was not possible with the current data to determine whether this factor was a contributor to the microbial alterations or a consequence of such changes.

Several reasons may explain the persistence of Bacteroidetes in the protein-deficient mice. A greater carbohydrate availability or lack of protein may favor bacteria that preferentially use carbohydrates or are less reliant on protein. Alternatively, such dietary variation could prevent competitors that favor protein fermentation from establishing. Verrucomicrobia (i.e., *A. muciniphila*) dominated the bacterial profile of 36-d-old mice fed either the dPD or dN diets for 13–14 d, which suggested that protein availability is not a major factor in the proliferation of *A. muciniphila*. This mucin-degrading bacterium can obtain its amino acids from the glycoproteins (e.g., mucins) that are produced by the host and has the capacity to catabolize all amino acids (16). Consistently, a positive correlation was shown between *A. muciniphila* and 3-indoxylsulfate. This metabolite arises from the breakdown of tryptophan in the gut by the intestinal bacteria, and tryptophanase, which is the enzyme involved in the metabolism of tryptophan, is represented by gene orthologs that are present in Proteobacteria, Bacteroidetes (Kyoto Encyclopedia of Genes and Genomes ortholog K01667), and *A. muciniphila*. By 46 d of age, this species decreased in the nourished animals, which indicated that the bloom in this mucin-degrading bacterium forms part of a natural colonization process that is independent of protein availability.

Amino acids are important carbon, nitrogen, and energy sources for the intestinal microbiota. Several amino acid catabolites that derive from the gut microbiota were excreted in lower amounts by protein-deficient mice than by well-nourished mice. These catabolites included *N*-phenylacetylglycine, isobutyrate, isovalerate, and the biogenic amines cadaverine and putrescine, which derive from the bacterial metabolism of phenylalanine, valine, leucine, lysine, and ornithine, respectively (17). The microbial genes involved in *N*-phenylacetylglycine metabolism are predominately present in members of Proteobacteria with other orthologs that are present in Firmicutes and Actinobacteria (Kyoto Encyclopedia of Genes and Genomes ortholog K01912) (18). Consistent with this involvement, Firmicutes and Proteobacteria were significantly lower in the dPD-fed mice than in the well-nourished mice. A change in the biochemical output of the microbiota after the dPD diet was not restricted to protein-derived substrates. Dimethylamine, trimethylamine, and trimethylamine-*N*-oxide, which are derived from the bacterial metabolism of dietary choline, were excreted in greater amounts by mice fed the dPD diet, whereas choline was excreted in lower amounts, than in the well-nourished mice. These observations imply a shift in the metabolic strategy by the gut microbiota toward choline use in the absence of protein, possibly with the use of choline as a carbon and nitrogen source. Because, choline was provided in all diets as choline bitartrate, the greater excretion of tartrate by the dPD-fed mice further supported this hypothesis. In addition, we recently reported a decrease in endogenous choline metabolism in undernourished children from Northeastern Brazil (4). Other microbial-mammalian cometabolites that were disrupted by the dPD diet included 2-hydroxyisobutyrate, hippurate, 4-hydroxyphenylacetate, *m*-hydroxyphenylpropionylsulfate, and cinnamate derivatives.

The protein-deficient diet has a notable impact on the energy strategy of the host. The higher carbohydrate content of the dPD diet (84.2%) compared with the dN diet (64.1%) resulted in the greater excretion of TCA cycle intermediates and sucrose. The greater excretion of *N*-methylnicotinamide and NAO in these mice may also have resulted from an upregulated TCA cycle through NAD(H) generation, which enters the nicotinate and nicotinamide pathway. Excess nicotinamide can be methylated to *N*-methylnicotinamide and further oxidized to 2-PY and 4-PY or NAO. Consistent with studies that fed rats a protein-free diet, dPD-fed mice excreted lower amounts of 2-PY and 4-PY. This result was previously shown to be due to decreased 2-PY and 4-PY aldehyde oxidase activity (19), and similar results have been reported in children (20). Thus, increased NAO excretion may be a consequence of reduced oxidase activity. In contrast, the lower excretion of hexanoylglycine, which is an intermediate of fatty acid β oxidation, by the protein-restricted mice indicated a downregulation of lipid oxidation despite having contained the same amount of fat as in the dN diet.

Moderate perturbations in the urinary metabolic phenotypes of zinc-deficient mice were observed compared with in the well-nourished animals. Zinc deficiency promotes protein degradation in muscle and reduces protein synthesis. These effects have the potential to reduce the BCAA pool (21) and are consistent with the reduced excretion of BCAA catabolites, 2-oxoisocaproate, 2-MOV, and 2-oxoisovalerate after consumption of this diet for 10 d. A downregulation of carbohydrate metabolism and lipid oxidation was implied by the lower excretion of 2-oxoglutarate, *N*-methylnicotinamide and NAO, and hexanoylglycine. Because zinc is an important cofactor for >300 enzymes, these observations may reflect impaired enzyme activity (22). In this study, only marginal alterations were noted in the fecal microbiota after zinc malnutrition. We previously showed that the consumption of a dZD diet for 2 wk significantly reduced zinc in the serum of mice but, to a lesser extent, in the luminal contents and intestinal tissue (14). It is plausible that the microbiota of the zinc-restricted mice may have obtained residual zinc from the host, thereby further depleting the host of zinc. Alternatively, zinc-associated bacterial alterations may occur at a lower taxonomic level than were measured here. In chickens, chronic zinc deficiency promoted a significant increase in Proteobacteria, which is a phylum that represented a major proportion of the fecal microbiota of dZD mice (23, 24). However, this phylum constituted a major proportion of the well-nourished profile of 46-d-old mice, increasing notably from 36 d old, and may, therefore, be part of the microbial aging process. This age-associated expansion of Proteobacteria may have obscured the increase associated with zinc deficiency. Under zinc-limiting conditions, several species within this phylum can induce the high-affinity zinc transporter ZnuABC (25), thereby conferring a growth advantage to these organisms that potentially explains their dominance in the dZD microbiome. Despite the mild disruptions observed in the microbial composition, a functional modification to this bacterial community was suggested by lower excretions of trimethylamine and dimethylamine in zinc-deficient mice. This result indicates a lower bacterial degradation of choline, which requires zinc for its demethylation, increasing its availability for the host.

In conclusion, these findings are consistent with metabolic alterations previously observed in malnourished children. The results show that we can model the metabolic consequences of malnutrition in the mouse to help dissect relevant pathways that are involved in the effects of undernutrition and their contribution to environmental enteric dysfunction. The potential for protein deficiency to influence the maturation of the gut microbiota represents another developmental impact of early life malnutrition. Because of the far-reaching influence of this microbial community, the importance of such perturbations on host development warrants further pursuit.

## Footnotes

7 Abbreviations used: BCAA, branched-chain amino acid; dN, defined normal; dPD, defined protein deficient; dZD, defined zinc deficient; HCA, hierarchical clustering analysis; NAO; nicotinamide-*N*-oxide; NMR, nuclear magnetic resonance; OPLS-DA, orthogonal projection to latent structures discriminant analysis; OTU, operational taxonomic unit; PCoA, principal coordinates analysis; TCA, tricarboxylic acid; 2-MOV, 3-methyl-2-oxovalerate; 2-PY, *N*-methyl-2-pyridone-5-carboxamide; 4-PY, *N*-methyl-4-pyridone-3-carboxamide.
